# The interplay between suicidal experiences, psychotic experiences and interpersonal relationships: a qualitative study

**DOI:** 10.1186/s12888-023-05164-2

**Published:** 2023-11-24

**Authors:** Patricia Gooding, Gillian Haddock, Kamelia Harris, Menita Asriah, Yvonne Awenat, Leanne Cook, Richard J. Drake, Richard Emsley, Charlotte Huggett, Steven Jones, Fiona Lobban, Paul Marshall, Daniel Pratt, Sarah Peters

**Affiliations:** 1 Division of Psychology and Mental Health, School of Health Sciences, Faculty of Biology, Medicine and Health, Manchester Academic Health Sciences Centre, University of Manchester, Coupland Building 1, Oxford Road, Manchester, M13 9PL UK; 2 Greater Manchester Mental Health NHS Foundation Trust, Manchester, UK; 3 Department of Biostatistics and Health Informatics, Institute of Psychiatry, Psychology and Neuroscience, Kings College London, London, UK; 4 Lancashire and South Cumbria, NHS Foundation Trust, Lancashire, UK; 5 Spectrum Centre for Mental Health Research, Division of Health Research, Faculty of Health and Medicine, Lancaster University, Lancaster, UK

**Keywords:** Non-affective psychosis, Schizophrenia, Interpersonal relationships, Suicidal experiences, Suicidality, Suicidal thoughts, Suicidal behaviours, Qualitative methods, Interviews

## Abstract

**Background:**

Suicidal thoughts, acts, plans and deaths are considerably more prevalent in people with non-affective psychosis, including schizophrenia, compared to the general population. Social isolation and interpersonal difficulties have been implicated in pathways which underpin suicidal experiences in people with severe mental health problems. However, the interactions between psychotic experiences, such as hallucinations and paranoia, suicidal experiences, and the presence, and indeed, absence of interpersonal relationships is poorly understood and insufficiently explored. The current study sought to contribute to this understanding.

**Methods:**

An inductive thematic analysis was conducted on transcripts of 22, individual, semi-structured interviews with adult participants who had both non-affective psychosis and recent suicidal experiences. A purposive sampling strategy was used. Trustworthiness of the analysis was assured with researcher triangulation.

**Results:**

Participants relayed both positive and negative experiences of interpersonal relationships. A novel conceptual model is presented reflecting a highly complex interplay between a range of different suicidal experiences, psychosis, and aspects of interpersonal relationships. Three themes fed into this interplay, depicting dynamics between perceptions of i. not mattering and mattering, ii. becoming disconnected from other people, and iii. constraints versus freedom associated with sharing suicidal and psychotic experiences with others.

**Conclusion:**

This study revealed a detailed insight into ways in which interpersonal relationships are perceived to interact with psychotic and suicidal experiences in ways that can be both beneficial and challenging. This is important from scientific and clinical perspectives for understanding the complex pathways involved in suicidal experiences.

**Trial registration:**

ClinicalTrials.gov (NCT03114917), 14^th^ April 2017. ISRCTN (reference ISRCTN17776666.); 5^th^ June 2017). Registration was recorded prior to participant recruitment commencing.

## Background

Suicidal experiences include, but are not limited to, thoughts, urges, compulsions, plans and attempts. They are invoked by, and associated with, immense psychological pain and distress [1–3]. Suicidal experiences are far more frequent than suicide fatalities and are strong predictors of death by suicide [4, 5]. The Adult Psychiatric Morbidity Survey stated that in 2014 in England, UK, 21.6% of individuals experienced suicidal thoughts, 6.9% attempted suicide and 0.01% died by suicide [6]. People who have severe mental health problems such as psychosis are more likely to take their own lives and have to contend with frequent, and sometimes unpredictable suicidal experiences [7]. Non-affective psychosis is often characterised by hallucinations, delusions and paranoid thoughts and feelings, with death by suicide being reported as having a standardised mortality ratio of over 12 for people with schizophrenia [8].

It is vital to understand the factors which are precursors to, and escalators of, suicidal experiences from numerous perspectives including those which are epidemiological, neuropharmacological and psychosocial. A research focus on the former two perspectives continues with a robust impetus [9–12] but less so on the latter. A psychosocial understanding of the mechanisms underpinning suicidal thoughts and acts is important scientifically but also clinically in that it provides an evidence-based foundation for developing efficacious and effective psychological therapies which can target suicide-related distress and prevent death by suicide [2, 13–15].

Several contemporary psychological models of suicidal thoughts and behaviours have highlighted the role of perceptions of negative social interactions and difficult social or interpersonal relationships [16–19]. The model which does this most explicitly is the Interpersonal Theory of Suicide in which ‘thwarted belongingness’ and feeling a burden play central roles [18–20]. There is an abundance of evidence demonstrating the deleterious impact of destructive social relationships, including those which are abusive, for people with different mental health problems [21–26]. Evidence is also accumulating in support of difficult social relationships intensifying suicidal thoughts and acts in people experiencing psychosis. For instance, perceptions of social isolation, being alone and the anticipation of being alone have been found to exacerbate suicidal ideation [27, 28]. In people with non-affective psychosis, experiences of not having social support were found to amplify suicidal thoughts because they increased feelings of defeat and entrapment [29]. This work is promising, however, three inter-related issues need to be probed with more precision.

First, social interactions are usually experienced as multi-faceted and culturally and contextually dependent [30]. It is unclear how effectively measures of interpersonal relationships reflect numerous aspects of discontent with social relationships (e.g., feeling criticised, controlled, disconnected, not understood, isolated and alienated). Relatedly, individuals who experience hallucinations can feel different types of interpersonal connections with, for example, hallucinatory voices and/or images. For such individuals, these kinds of connections with hallucinations may seem very ‘real’ and can have similarities with the complexities of social connections with the people who form their social networks. Similarly, paranoia and other delusions can be social/interpersonal constructions [31–34]. The interplay between these types of interpersonal connections and suicidal experiences is insufficiently understood. Qualitative methodologies are best suited for addressing these limitations because they are based on naturalistic participant accounts.

Second, it is important not only to have a transdiagnostic approach to delineating the pathways underpinning suicidal experiences as is the case for most contemporary psychological models [14], but also to expand these models so that they incorporate the effects of specific mental health problems within those pathways [13]. In particular, we need to have a more comprehensive understanding of the ways in which social situations can affect experiences such as hearing voices and/or feeling watched, and how this impacts suicidal thoughts and behaviours [27, 28, 35, 36].

Third, there is a pressing need for suicide prevention approaches to investigate and appreciate ways in which people with severe mental health problems are able to counteract suicidal experiences [37, 38]. This area is growing and includes epidemiological [39] and psychosocial research initiatives [16, 40–45]. A recent review examining the components of resilience in people with psychosis identified five factors pertaining to social interpersonal relationships which were: actively seeking support from within communities and other people (e.g., family and mental health professionals); having a sense of meaning from religious beliefs; having reasons for living; having an awareness of personal skills including self-esteem; and being able to cope with emotional lability [41]. Despite progress, work in this area requires robust expansion with a particular focus on how individuals with psychosis understand the complex dynamics of interpersonal relationships, social communication, and a broader sense of social identity (e.g., belonging to a community) in the context of living with suicidal thoughts and behaviours.

## Method

### Aim

The overarching aim of the current study was to investigate the interplay between perceptions of social interpersonal relationships, non-affective psychosis, and suicidal experiences in people currently living with these severe mental health problems [13]. A qualitative approach was used in which individuals were interviewed in a one-to-one setting in order to nurture an in-depth understanding of the complexities and dynamics of interpersonal relationships in the context of suicidal experiences and psychosis.

### Patient and public involvement

A group of Experts-By-Experience (EBEs) advised on all stages of the research process comprising the current study, including the training of research staff. The EBEs had all experienced severe mental health problems such as psychosis and suicidal thoughts and acts.

### Design

A thematic analysis was conducted on transcripts from semi-structured interviews. The data were collected as part of a two-armed (treatment versus control) randomised controlled trial (RCT) called Cognitive AppRoaches to coMbatting Suicidality [CARMS] [13].

### Participants

Participants lived in the community or were on inpatient psychiatric hospital wards across the Northwest of England, UK. Referrals were made into the CARMS trial mainly via mental health care teams primarily from three National Health Service (NHS) Trusts which were Greater Manchester Mental Health NHS Foundation Trust, Lancashire and South Cumbria NHS Foundation Trust and Pennine Care NHS Foundation Trust [13].

### Eligibility criteria

Inclusion criteria were: i. meeting International Classification of Diseases, 10^th^ edition (ICD-10) criteria for non-affective psychosis (F20-F29) including schizotypal and schizoaffective disorders [46]; ii. self-reported suicidal experiences, including thoughts, urges, plans, acts and/or attempts in the three months prior to recruitment into CARMS; iii. participants aged 18 years or older; iv. English speakers or having sufficient competency of the English language not to need an interpreter to participate; v. under the care of a NHS mental health team with a care co-ordinator; and vi. able to give informed consent in accordance with the British Psychological Society’s (BPS) guidelines (http://www.bps.org.uk/sites/default/files/documents/code_of_human_research_ethics.pdf). The exclusion criteria were i. ICD-10 dementia or organic brain disorder diagnosis; and ii. currently participating in a psychological intervention clinical trial.

### Sampling and selection

CARMS participants were invited to take part in a qualitative study about their experiences of suicide and psychosis. Interviews took place either on entry to the CARMS trial, or at the 6- or 12-month follow-up time points [13]. Participants for the current study were selected from those who expressed an interest using purposive sampling defined by a sampling matrix [47]. Age, treatment allocation, time point of interview (baseline, 6 or 12 months), and relationship status were identified in the matrix to maximise richness and variation in the data. Furthermore, as the overall study aim was about suicidal experiences, it was important to sample participants with a range of scores on baseline measures of suicidal thoughts and attempts. Additional clinical information which was collected from participants is documented in the CARMS trial protocol and included general psychiatric symptoms, positive and negative psychotic symptoms, drug and alcohol use, and, dependent on allocation condition, therapy process variables (e.g., number of therapy sessions, time in therapy) [13].

### Suicide ideation measure

The Beck Scale for Suicidal Ideation (BSS) [48] is a 21 item self-report measure of severity of suicidal ideation (items 1–19) and history and severity of suicide attempts (items 20 and 21) with each item being scored 0, 1 or 2. The ideation items are designed to capture a range of suicidal thoughts of graduated severity and include active suicidal desire (e.g., “Do you ever have the desire to kill yourself”), passive suicidal intent (e.g., “Would you try to save yourself if you found yourself in a life threatening situation?”, planning (“Have you made preparations?”), intensity (“If you do think about killing yourself, how often do you think about it?”) and deterrents (“Do concerns about friends and family, religion or possible injury from an unsuccessful attempt stop you?”). Cronbach’s alpha inter-rater reliability coefficients were reported as 0.90 and 0.87 for psychiatric inpatients and outpatients, respectively.

### Procedure

Participants provided consent to take part in the current study. Individual qualitative interviews were conducted by a researcher face-to-face at participants’ homes or in NHS Trust mental health facilities. Interviews were audio-recorded, using an encrypted audio recording device, transcribed verbatim by a professional transcription company, and checked for accuracy by the researcher. Participants were financially compensated with £10 as a ‘thank you’ for their time. Interviews followed a flexible topic guide initially developed from previous suicide-related work within the research team; the guidance of EBEs; and relevant published suicide literature [49]. During the interviews, participants were invited to share their experiences of mental health problems and different aspects of their suicidal experiences including how such experiences seemed to be triggered and/or escalated, together with the converse, that is, any factors which seemed to counter these types of experiences. It should be noted that participants were not asked about the dynamic interactions between their interpersonal relationships and suicidal experiences directly. Rather, this was further probed if it arose as part of exploring pathways to suicidal experiences.

### Ethical issues

The current study was approved by a National Health Service (NHS) Research Ethics Committee (reference: 17/NW/0089). Relevant ethical and legal guidelines concerning confidentiality and the storage of personal data were followed (e.g., General Data Protection Regulation). Signed informed consent forms were stored on NHS and/or university secure servers, separate to the research data. Participants provided informed consent for quotes to be used in publications. Transcripts were anonymised before being analysed. For example, identifiers replaced proper nouns (such as names of people, buildings, places). Anonymised transcripts were stored electronically on password protected files on a university system and accessed remotely from personal computers only through the university virtual private network. The CARMS RCT was registered on ClinicalTrials.gov (NCT03114917) on 14^th^ April 2017. Registration took place prior to the first randomised participant.

### Data analysis

Data were analysed using inductive reflexive thematic analysis (TA) at a sematic level with an interpretative stance. A critical realist perspective was adopted, whereby it was assumed that participants’ accounts reflected their subjective reality [50, 51]. The data analysis was conducted by M.A. under the supervision of S.P. (an expert in qualitative research methods) and P.G. (an expert in suicide). Initially, M.A., P.G. and S.P. independently coded, and discussed ensuing interpretations of three transcripts. Thereafter, researcher triangulation was employed to maximise the trustworthiness of the final analysis whereby regular discussions between M.A., S.P. and P.G. enabled a range of perspectives and interpretations to be considered and thus mitigate any potential biases of a single researcher [47].

The procedure outlined by Braun and Clarke [50] was followed throughout the analysis which included: i. initial familiarisation by reading each transcript several times noting significant statements and keywords; ii. line by line coding to develop candidate codes; iii. code refinement; iv. code revision, organisation, and grouping in order to address the research aim; and v. auditing of each theme to ensure that they were independent of other themes. An iterative approach was taken to establish data sufficiency whereby transcripts and codes were repeatedly reviewed and discussed with the supervisory team until no additional codes or themes were identified [52]. The final analysis and accompanying narrative were discussed and refined with input from the wider research team to select the final choice of illustrative quotes and the conceptual model. The data and analytic processes were organised using the software package NVivo version 12 (REF).

## Results

### Participant characteristics

Interviews with 22 participants were included in the current study. Fifty per cent were assigned to the treatment arm of the trial (one was assigned to the treatment condition but did not receive any therapy sessions for logistical reasons). The mean number of therapy sessions received by these 11 participants was 17.64 (SD = 6.89, Range = 0–24). The mean age was 37.6 years (SD = 13.9, range = 18–62). Eleven were female and 11 were male. Nineteen identified as White/Caucasian; 16 were single; and 13 reported living alone. Interviews took place between 1^st^ November 2017 and 25^th^ February 2020 and lasted on average 43 min (SD = 15.4, Range = 16–76 min), with three lasting over an hour. Suicidal ideation scores, as measured by the Beck Scale for Suicidal Ideation [BSS] items 1 to 19 [48], ranged from 5 to 24 (Mean = 16.23; SD = 6.0; possible range 0–38). Fourteen participants had made more than one suicide attempt throughout their life and five had made one attempt. For 13 participants, their wish to die when they made their last attempt was high and for six it was moderate. Eighteen participants had a diagnosis of schizophrenia, three of schizoaffective disorder, and one had a diagnosis of persistent delusional disorder. Over the past three months, six participants reported using drugs and 13 reported using alcohol, with four using both drugs and alcohol.

### Overview of key findings

**Fig. 1:**
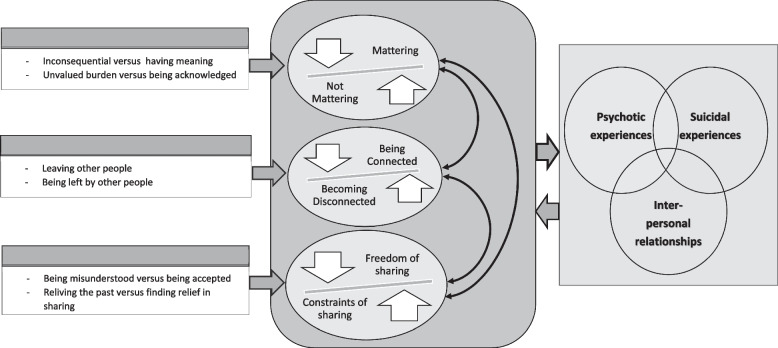
Conceptual model of the interpersonal social dynamics in the interplay between suicidal experiences, psychotic experiences, and experiencing other people

Across participants, there was an overall context reflecting a complex, multi-directional interplay between psychosis, suicidal experiences, and interpersonal experiences. This interaction was influenced by processes that are described within three themes: i. Not mattering and mattering, ii. Being connected and becoming disconnected, and iii Constraints versus freedom. Each theme was organised into two sub-themes forming a novel conceptual model (see Fig. 1).

### Overall context: an interplay between suicidal experiences, psychotic experiences, and interpersonal experiences

Suicidal experiences were sometimes described as thoughts and/or plans but at other times they seemed less like a thought and more akin to being overwhelmed or consumed. Feeling overwhelmed or consumed could happen rapidly and without warning. Participants described how psychotic experiences, for example paranoid feelings and hallucinatory voices, were central to them self-harming, feeling suicidal, and attempting to kill themselves. Hallucinatory voices were intensely negative for all participants, one of whom relayed an instance where the voice encouraged them to stop being alive: “*They tell me to–, that I'm not worthwhile and–, I should just do the world a favour and stop breathing… I agree with them.” (P13).* Simply being in the presence of individuals, including those who were unknown to participants could affect their state of mind, largely, negatively. Whilst being with other people, for instance by seeing them or hearing them talk, could amplify some psychotic experiences, for example suspiciousness, the effect of other types of psychotic experiences, such as negative hallucinatory voices, could be made worse by being alone:

*“… and I was hearing a voice, a few voices, telling me to handle a knife, and I ended up picking it up and putting it to my throat… I was struggling all day. No one was in the house. It was just me, and I was having a tough day.” (P19).*

One person expanded on the reason why the voices wanted them to kill themselves which starkly characterised how they felt other people viewed them *“‘Cause they don’t want–, ‘cause they don’t like me and they’re just representing, like, what everybody else thinks.” (P4).* Explanations by participants captured the ways in which hallucinatory voices could seem a very real presence and how they could become harrowingly aggressive if they tried to talk to other people about their voices. These types of experiences brought with them feelings of immense threat and of being constantly beaten down:

*“I don’t know what it’s like [long pause], they’re [the voices] screaming at me [short pause]. ‘Don’t you dare tell them anything’, they’ll kick off even more now ‘cause I’m telling you what they’re saying, […] you can’t win. I’ve got a man and a woman, the woman’s in my head and the man’s out of my head, I had the woman--, I had the man first--, when I had the first breakdown, I got the man, and he was out of my head. It was like having someone in the room with me, it was so scary. And when I first heard him, the first thing he ever said to me was ‘Jump in front of that bus.’” (P8).*

This participant had thought about killing themselves every day and had made numerous suicide attempts in the past because of hallucinations, including one about eight weeks prior to their interview. They described how Satan, who had appeared in a visual hallucination, had caused them to become friendless: “*When, when Satan came to me [in year], he messed up all my Facebook, making me type out all this stuff, things. All my friends left me, you know.” (P8).* This social abandonment and isolation was echoed by other participants who indicated that voices stopped them from going out and meeting new people. This could be enacted by explicit instructions from voices; fear of the consequences of being with friends or family if the voices became angry; feeling that understanding was not possible from other people, including mental health professionals; and facing judgement and stigma from friends because of psychosis. All these fears fed into the desire to end their life.

### Theme 1: Not mattering and mattering

The first theme encapsulated thoughts and emotions of participants which portrayed not mattering and mattering. For some, mattering was on a continuum whilst, for others, it was more binary. Participants felt that they did not matter to themselves, nor to others, in different ways. For example, they felt that they were unimportant and that they would not be missed if they were to disappear or die. At the opposite end of this continuum, others reported a realisation that they were important to others and that they did have a role which imparted meaning to their own life. Some felt that they were a burden to others around them, and that people close to them would be freer if they, the participant, were to die. Again, there was an opposite side to these thoughts, evaluations and feelings which was an almost overriding sense of worth when participants perceived that they were explicitly acknowledged, valued, and appreciated and that they were not considered a burden. Therefore, these perceptions have been represented along two continua in sub-themes of: i. Feeling inconsequential versus having meaning; and ii. Feeling an unvalued burden versus being acknowledged and valued.

#### Sub-theme i: Feeling inconsequential versus having meaning

Participants relayed a personal sense of not mattering and of being irrelevant to other people. Their suicidal thoughts and feelings were rooted in this inconsequentiality. This led them to feel that, if they killed themselves, then their death, their absence, would be of no importance to the people around them; it would be insignificant or trivial. One participant explained that they felt as though they were an outcast because they had not succeeded at anything which had led them to think that they could not relate to anyone and of not wanting to be connected to other people:

*“Well, I feel like an outcast. That's how I feel… in my own little world. That's how I feel… I'm just, I'm just… I think life itself, I'm a little bit fed up with life itself. And since I've had enough. I don’t really feel--, like I need to, I want to get involved in--, people [short pause]. No.” (P15).*

However, juxtaposed with these impressions and feelings of being irrelevant was a thought process which, as it developed, conveyed the opposite, and served as a counter. It captured the obverse of feelings of inconsequentiality and underscored to some participants that they did matter to others. Moreover, it highlighted that they had made an impact on the lives of those around them and this contributed to giving them a sense that their own life had meaning. A number of participants expressed inconsequentiality, and its opposite of mattering, not necessarily as a concrete binary but more as a continuum reflecting fluctuation and change:

*“One of the main things that was around killing myself, it's like, if I kill myself, who's going to miss me? Who's it going to affect? And the more you think about that actually, I've got my mum, I've got my dad, I've got my sister, I've got the animals. I've got all the people that I've met, all the people that I've spoken to. All the people that I've made a decent impact on. They don’t really know me, but I've helped their life. And it's massive when you think about it like that, I think […].” (P20)*

Some participants communicated how having other people in their lives could change their views about death in general which was broader than suicide: *“I would, essentially, happily just cease to exist. But I’ve got other people in my life and it’s not just about me.” (P12)*. This carried with it a sense of movement, going from being resigned about death and having no regrets about dying, to a change wherein participants described having a commitment to a relationship and associated with that a perception of a future that held promise. Some participants identified a change in themselves whereby they did not want to die which had come about from forming personal relationships:

*“Well, my outlook on death has changed. I’ve always said that if I died tomorrow, I’d be happy because I’ve done enough in my life and, you know, I’ve got no regrets. But since the engagement, I don’t want to die now. I want to grow old with [partner]. So, it’s funny how just a commitment has totally changed my outlook on my future. […] if my time came, then that would be it, do you know what I mean, and that I wouldn’t bother, but now--, say like if I got cancer for example, it wouldn’t scare me, death wouldn’t have scared me, but if I got cancer now, then I don’t want to live without [partner], you know, I want a future with him. Life’s just starting again for me.” (P26).*

#### Sub-theme ii: Feeling an unvalued burden versus being acknowledged

In this second sub-theme, participants expressed feeling alienated from people because they believed that they were bothering them and were a burden to them: *“It [speaking with care-coordinator] just to touch base with someone. Other than my mum ‘cause I feel like I’m bothering her, so.” (P28)*. One person articulated a perception that if they did kill themselves, then they felt that those close to them would be better off without them because they saw themselves as unable to help them: *“What happens if I killed myself and then I die myself, and then maybe there is no problem with my niece anymore.” (P7)*. However, this perception was not necessarily fixed and could be more fluid. For instance, in the same interview, this participant said that they reasoned with themselves, coming to the conclusion that they could, in fact, help their niece, and because of this, felt that they had something to value in their life.

A consequence of feeling a burden was that individuals felt undeserving of help from others *“[short pause], well she's [therapist] on about, [short pause], the support I get and… it felt like she was saying that I don’t deserve the support.” (P13)*. Talking about their psychotic experiences was observed as potentially troublesome and onerous to other people, especially if it became intermingled with the other person’s mental health problems. Similarly, if people around them seemed able to take on-board the participant’s mental health problems, then participants worried that this represented unwanted ‘baggage’ and could detrimentally change the way that they interacted with those individuals in the future:

*“the more I do see people, the more they get to know me, then maybe it's not so good. Well, in a way it would be good, but then they take all the baggage with, that comes with me, with them. In fact, that was something I did discuss with [name 1] was that, er, often I go to somewhere where everyone was really… kind and polite, and everything, and I've been worried in case the next time I went that they wouldn’t be like that.” (P10).*

The reverse side of this issue was a sense of support in being able to talk to someone, for instance a mental health professional, who participants believed would not get overwhelmed by the participant’s problems: “*And it was useful, it was really good. It's… it's having someone to speak to that doesn’t get completely and utterly boggled by what I'm talking about.*” *(P20).* Implicit in this last quote is an implication that many people do not seem to be able to understand psychotic experiences, or feel comfortable being a witness to those experiences. Hence, finding someone who is able to be with psychotic experiences can feel supportive.

In contrast to feeling undervalued, being acknowledged and appreciated by other people was pivotal to how some participants countered their suicidal thoughts and feelings. This message of acknowledgement and acceptance was received from friends and family but also from mental health professionals: *“She [care coordinator] just lets me talk freely and doesn’t judge me, and doesn’t ask too many questions about why this, why that.” (P4).* Participants explained that having family members who recognised what they were going through and were accepting and non-judgemental made them feel understood, acknowledged and cared for. Furthermore, for some, there was a difference between friends and family in how they communicated value:

*“so, I know if I went to, like, a family meal or something, then and if I’d, like, literally just got out of bed, they’re not going to judge me for it. Whereas if you went and saw your friends, and you’d just got out of bed, they’d mention it straight away.” (P21)*.

Perceptions of having a specific role in relationships, for example, being a parent, was another way in which participants gained a sense of purpose which offset suicidal desires: *“But no one leave me alone long enough for me to ever get away with it [suicide], which I'm glad of 'cause I've got a son.” (P12)*. Some participants actively reflected on the support they provided for their children which, in turn, made them feel needed, and that their life had a purpose. The following participant underscored the importance of this kind of role with a parallel perception that they had no one else in their lives apart from their children:

*“That's what keeps me going, my kids, yeah. I've got no other family. I've got no… real friends round here. And that's it, my kids, yeah. And I, I do look after them. That's all that's left of me now.” (P17).*

Stigma and intolerance towards mental health problems generated feelings of being dismissed and not being valued. Whilst some had the experience of interrelationships with other people, in particular family members and mental health professionals, conferring a sense of being valued, for others, stigma and judgement were detected from friends, family, and mental health professionals:

*“Like one appointment she [a previous care co-ordinator] was two hours late and it wasn’t even in my house, I wasn’t warm, it was in the middle of winter, I was outside, it was a coffee shop waiting for her to turn up and then so after I phoned about three times she turned up. So and I felt sort of judged by her.” (P4).*

One person explained how, in their view, having mental health problems was seen as a weakness. Moreover, for this participant, seeking help because of mental health problems was also seen as a weakness:

*“Showing emotions is, or was, considered a massive sign of weakness, and asking for help was a sign of weakness. And everything that could help you get better was a sign of weakness. So, I never did any of it.” (P2)*.

The influences of stigma illustrate the ways in which prejudiced, judgemental and intolerant views held by others about mental health problems added to self-perceptions of being unworthy, undeserving and a burden, and relatedly, added to self-stigma. An additional, negative layer of self-appraisals took hold when individuals themselves absorbed and then applied those intolerant views to themselves resulting in a reluctance to seek help.

### Theme 2: Being connected and becoming disconnected

This second theme revolved around thoughts and feelings about becoming disconnected from other people and living with the loss of others including experiencing loneliness and emptiness*: “Loss of a loved one. What’s the purpose of living if you’ve lost the person you love?” (P26).* These disconnections arose for different reasons and within different, albeit overlapping, contexts. Examples included, thinking of the consequences of leaving other people because of suicide, being reluctant to connect with others from the outset, actively disconnecting from relationships after they had been formed, experiencing others breaking off relationships, and being bereaved. These different types of disconnection could interact with psychotic experiences and also be part of the pathways to suicidality. Therefore, two sub-themes were developed to represent these diverse circumstances which were: i. Leaving other people, and ii. Being left by other people.

#### Sub-theme i: Leaving other people

This sub-theme described two ways that participants felt that they left other people. The first captured thoughts and feelings about leaving people behind after dying by suicide. The second captured situations surrounding a choice to break off relationships and friendships with other people.

It must be emphasised that all participants described wanting to die by suicide because they felt they had reached their limit of dealing with the extreme and diverse difficulties, fear, and sheer exhaustion which they encountered every day of their lives and which, for many, had been persistent over many years. For example, one participant was molested as a child by her father and experienced explicit images of that abuse which they believed had been put into their head by Satan. They stated: *“I've been fighting it for the last eight years and I'm exhausted” (P11)*. It is from these kinds of circumstances that the people we interviewed had come to have suicidal experiences. Within those contexts, some participants had thoughts about their loved ones facing their death by suicide which stopped them making an attempt: “*Sometimes I’ve had a knife but then I think of my sister, like, I throw the knife away.” (P1).* Some experienced guilt at the thought of the effect that their suicide would have on other people: “*I felt guilty… Because… I felt guilty for me family.” (P3).* Some people actively evoked these types of thoughts as a deterrent to attempting suicide: “*And I took that to the extremes and envisioned what it would be like to see my sister standing at my grave crying. And that was one of the reasons why I'm still here.” (P20)*.

Participants described that when they had suicidal experiences, they often felt a total disconnection from others. Sometimes this was because they made a choice not to be with people. For example, they reported experiencing debilitating anxiety and/or panic attacks alongside psychosis with a bi-directional link between psychotic experiences and anxiety which served to dissuade them from interacting with other people:

“*Just they, they [anxiety and voices] normally come together. Like they used to always come together. It would be more something in my head telling me somebody’s watching me. And then that sets off my anxiety, and then I’m a mess.” (P12).*

Hence, making contact with other people socially, for instance, with friends or family, was perceived as difficult, if not impossible. This was escalated further if social situations involved new people. This meant that some participants made a choice that seemed less threatening for them which was not to meet or make contact with others. More generally, being with, or even just seeing others could strengthen feelings of being watched. This could then make people to want to be alone. Furthermore, choosing to disconnect because it gave a sense of space from mental health problems was not necessarily viewed negatively by the people we interviewed. For example, one person explained that, although they felt that being alone was not ideal, it was felt like it was the right decision at the time because they had been hospitalised due to mental health problems including suicidal experiences: *“So, to just disconnect yourself for a bit and not make any decisions is like, the better option.” (P21)*. Another participant described a slightly different experience which was a desire to be alone, to not socialise, but weighing against that were thoughts that having social contact was an important part of daily life:

*“I guess I'm starting to see how… my problems affect me with social, actual social things... I've noticed it from young, but you know I, you know, it's more daunting when you get older 'cause you actually realise. ‘Cause socialising is a part of everything, really, you can't be alone all the time. I know it would be nice, but you know.” (P27).*

In general, feeling disconnected from other people was viewed as unwanted, troubling, and inadequate. That said, a sense of disconnectedness could arise for many reasons, some of which seemed necessary and advantageous, at least in the short-term.

#### Sub-theme ii: Being left by other people

This sub-theme portrayed the experiences of participants when facing the loss of different types of relationships wherein they had little or no control. The first type of loss was the death of a loved one: *“I went through a bad patch where I was pregnant, and I lost my daughter.” (P12).* Participants described the struggle which they lived through and which for some did not really abate after the death of someone close to them. For example, one participant relayed how the loss they felt when a caregiver had died, with whom they had a close and supportive relationship, could trigger them to start to become unwell with psychosis:

*“well what I think what, er, probably triggered it off was that my mom passed away. And that seemed to be, that seemed to be the thing like gave me that sort of, you know that push.” (P7).*

Losing important relationships was not just confined to the death of people with whom participants felt close. The second type of loss included friendships in which struggling with the effects of that loss was, for some, associated with increased suicidal thoughts and attempts:

*“I’d had a fallout with a friend, I thought, ‘Fuck this, I can’t be arsed anymore’, and I weren’t allowed to see my Godchildren anymore, so I just tried ending my life.” (P8).*

Despite finding it challenging to trust other people, some participants recognised how important trust was to a relationship. This also applied to the therapeutic relationship. Consequently, it could be exceptionally challenging for participants to negotiate therapeutic relationships coming to an end, even though those endings were expected. A feeling of disappointment and despondency could arise when the talking came to an end and the company of the therapist was no longer there:* “in a mad way a bit of company for me. Someone to talk to, yeah. So that that went away as well. All those things that I trusted him with.” (P5).* Some participants reported having had an abrupt ending to a therapeutic relationship and portrayed this as a type of rejection, experienced as abandonment:

*“And she [therapist] read it [a book the participant had written], and said, ‘You inspired me, but I can't work with you anymore because it's not my field [abuse].’ So I was devastated again.” (P5).*

In the context of a therapeutic relationship, a third potential loss came with the idea of recovery from voice hearing, which, despite wishing to stop experiencing hallucinatory voices, could leave an individual feeling abandoned and/or lonely. This was not reported as an experience but a hypothetical concern: *“I'd like to be rid of the voices. Although my therapist made a good point, she's like, ‘You've had them so long that you might feel lonely if you don’t hear them anymore.’” (P13)*. This highlighted the complex, interpersonal relationships participants could have with their voices.

### Theme 3: Constraints versus freedom

Constraints versus freedom reflected the consequences of talking and sharing experiences of suicide and psychosis. An important point should be emphasised at the outset which is an acknowledgment of how hard it was for participants to talk about the mental health difficulties, particularly suicide, that they faced in their lives. Within that context, this theme portrayed a dynamic relationship. On the one hand, a comfort in having human contact and being able to talk to other people about suicidal and psychotic experiences, and on the other hand, anticipated consequences of sharing suicidal and psychotic experiences and having to negotiate complex conversations, attitudes and beliefs:

*“I don’t talk to anyone about things regarding my BPD [Borderline Personality Disorder] and my delusions, my paranoia. So… like, friends or anyone, even my sister who I'm the closest with, I won't talk to any of them about that because it's gets, it causes uproars and things like that.” (P14).*

Consequently, this theme has been organised into two sub-themes, each of which reflects opposing experiences of talking and sharing, namely: i. Being misunderstood versus being accepted, and ii. Reliving versus finding relief.

#### Sub-theme i: Being misunderstood versus being accepted

Participants had very positive experiences when they felt genuinely listened to, accepted, and given the freedom to express their thoughts, feelings, and circumstances around psychosis and suicidality as they lived them. Being genuinely listened to was expanded upon by participants to an appreciation of having a truly ‘safe space’ in which to express themselves. This sense of safety was experienced when working with therapists: *“And it's just like, well, I think it's just because there's a level of safety and understanding that someone, well, you know understands, you're in this profession…” (P14).* The antithesis of this was situations in which participants did not feel safe. One of these was a fear that, if they talked openly about their suicidal thoughts, feelings, and plans, they would be involuntarily detained using the mental health act (i.e., ‘sectioned’). This fear was most evident when talking to mental health professionals:

*“I wasn’t able to voice that [talking about their suicidal thoughts]. I didn’t find the strength for saying that I felt like it was a weakness on my behalf. I was scared I was going to get sectioned. I was scared that, you know, they're going to, ‘Oh my gosh, she’s a nut job, let’s put her away.’” (P14).*

Participants also described how, despite the many occasions when they had felt listened to, there were many times when the converse had been the case. This happened during conversations with family and/or friends and with mental health professionals: *“I just wish somebody would listen for once in my life. I'm not lying, they put me through abuse.” (P5).* When this participant did not feel listened to by health and social care professionals, or the police, they attempted to deal with this by drinking heavily: *“After talking to the police last year, I put on nearly three stone. I was drinking three bottles of sherry a day because they didn’t listen.” (P5).* A related observation was that when people were in suicidal crisis, then that was a time when health professionals were less likely to really listen to them because they were occupied with assessing risk. This should be remedied because it was a time when genuine listening was paramount.

Feeling misunderstood or poorly understood by people who were, perhaps, expected to have a meaningful understanding could also act as a stimulus which worsened the state of mind of some participants. Agitation and frustration escalated as a consequence:

*“And they just kind of trigger you even worse. And with friends and my boyfriend, there's just no understanding, there's just--. There's just like, ‘Oh, I've had that once,’ you know, I was walking down the street and I thought--, I was like, ‘Oh right, that's very different to what I'm experiencing right now.’ Ah, you know, I've had to deal with for a long, long time. And they try, and it's lovely that they try and… relate in that way, but then you're placed with comparisons that feel like you’re agitated even further and triggered off worse.” (P14).*

Individuals explained that, rather than listening and attempting to understand them, there was often a tendency for others to give advice or make suggestions which was not founded on knowledge or experience of what it was like to live with psychosis and suicidal thoughts. On such occasions, participants reported not feeling listened to and understood by others and perceived talking and sharing as unhelpful and distressing:

*“It’s like, my mum’s like, ‘Tell them to go away’, it’s like, ‘Yeah, you try having voices in your head and you try telling them to go away when they’re in your head’. You’ve got one in your head and one out your head, do you know what I mean?” (P8).*

When interacting with mental health professionals, for example, their care co-ordinator, interviewees expressed sometimes feeling pressurised to conform to ideas that were not in line with their own conceptualisation and views of their mental health. Having a different perspective from mental health professionals and feeling that a minimal effort was being made to appreciate what they were saying, caused interactions to be difficult rather than supportive:

*“Not very well, we didn’t get on. […] Because I didn’t agree with what she said and she didn’t agree with what I said. She tried to make me conform to her answers, to as to why I hear voices and I didn’t agree with her” (P29).*

#### Sub-theme ii: Reliving the past versus finding relief in sharing

Participants’ perceptions about talking about suicidal and psychotic experiences had multiple components. These components included the negatives of reliving memories, which contrasted with relief of being able to share in this way. Again, for some participants, this seemed to be experienced as a continuum, whereas for others, it was more polarised. Participants told us how, during therapy, painful memories could be revisited, even when relaying thoughts and feelings about present-day life. For some, this led to feeling distressed both at the time of sharing and afterwards:

*“I think sometimes it [therapy] can trigger the thoughts, ‘cause you say you’re having an okay day and the therapy comes along and you have to talk about it, it might make your day much worse because you’re bringing back all them thoughts that are making you want to do that [attempt suicide].” (P4).*

However, this was also viewed by some as an inevitable and acceptable aspect of therapy whereby psychological pain was found to be offset by the release and relief felt because of talking: *“I mean, I understand for some people they could be triggering, but as I said, a lot more–, we need to talk a lot more about this rather than say, pushing it to one side.” (P17)*. One participant explained how being part of an online group which shared experiences of mental health problems was triggering for them but was also balanced by feeling beneficial:

*“I found a friend online, there was a group online that sort of half of the time it triggered me 'cause they were going through similar things to what I was going through. But half of the time they sort of helped me as well.” (P13).*

Participants reported that talking about suicidal experiences was often perceived by others to be unacceptable: *“Because… no one ever thinks about it or talks about it [long pause], because people don’t want to know.” (P20),* and that such thoughts should be kept hidden: *“‘Cos it’s [suicide] not something you talk about, really. You tend to keep it to yourself” (P22*). In contrast, participants indicated numerous times when talking about mental health problems and suicidal experiences was exceptionally beneficial and provided a sense of relief: *“It’s nice to get it off my chest” (P14),* even though it could be frightening:* “ it's scary. But you've got to talk about these things to help yourself.” (P18)*. Talking and sharing thoughts and feelings about suicide went further in that it was sometimes a way of regaining control from the hijacking effect of mental health problems: *“It helped me… ‘Cos when I'm in control I don’t feel as ill.” (P18)*. Moreover, talking and sharing was identified and recognised as an important part of coming to think differently about suicidal thoughts and acts that had the potential to be transformative: *“it’s just, actually, I mean you talk about it [suicide], I guess you think about it again and, you know you actually think it is, I guess stupid, I guess in certain ways” (P27).* That said, whilst talking and sharing frequently countered psychotic and suicidal experiences, this was not necessarily the case when participants had suicidal thoughts with high levels of intent. Participants explained that when they were exceptionally determined to make an attempt on their life, they were reluctant to share their thoughts and feelings because they believed that talking would not change their decisions:

*“But I don’t know--, the problem is with committing suicide, although you might think about it and plan it, the moment you actually do it, there’s no time to intervene, you just make a decision… If there was some sort of service--, like would I have thought, I’m trying to stop my heart, should I phone somewhere and ask for some advice? Because I was so determined, I don’t think I would have, I’d just go ahead with it.” (P26).*

The potential ineffectiveness of talking and sharing was also observed when participants spoke about not seeing any changes in their level of suicidality following their interactions with others. They stated that, despite talking to many people about their difficulties, they did not experience any improvements and felt that they were not achieving anything by sharing their problems. On such occasions, talking and sharing about suicide was seen as ineffectual:

*“I have an impression that I speak to various people about my problems that I have. But… in lots of ways I don’t seem to be getting any--, I don’t really seem to be getting anywhere.” (P7).*

In sum, although participants expanded on the many benefits to them of talking about, and sharing, psychotic and suicidal experiences, there could be perceived negative consequences which inhibited them from sharing in this way in the future. That said, sharing perceptions, thoughts and feelings served fundamentally to change how some individuals came to view suicide.

## Discussion

This is the first study to examine the tripartite interplay between psychotic experiences, suicidal experiences, and interpersonal relationships, using qualitative methods. A novel conceptual model (see Fig. 1) is presented of the mechanisms contributing to this interaction which comprised three themes, namely, perceptions of not mattering and mattering; connectedness and disconnectedness arising from leaving other people and/or being left by others; and both the constraints and freedom associated with talking about and sharing suicidal and psychotic experiences with other people. Integral to this model was its highly interactive and interlinked nature. For example, very present hallucinatory voices encouraged individuals to take their own lives whilst inculcating self-perceptions of being worthless and alone. The sense of a communicative and dynamic presence accompanying participants’ hallucinations resonated with a literature illustrating ways in which interpersonal relationships with hallucinatory voices and images share numerous similarities with ‘real’ interpersonal relationships [31–34]. Voices and images could intimidate individuals so that they felt not only unable to communicate with family, friends and mental health professionals but that they should deter those people from making contact with them. The effects of these types of psychotic experiences resulted in feelings of being constantly alienated, trapped, and beaten down, all of which are key precursors to suicidal thoughts and acts [53–56]. Work which has examined interactions between psychosis, suicidal experiences and interrelationships with other people is still relatively minimal. Examples of recent studies which have attempted to redress this limitation have focused on social problem solving [57], social behaviours using smartphones [58], social cognitive biases [59], and what was termed social dysfunction [60] using quantitative techniques. The model presented here reflects lived experiences which go beyond delineating simple continua, linear relationships or pathways.

The first theme in the conceptual model captured appraisals of not mattering and, conversely, appraisals of mattering, which for some existed on a continuum. Mattering has been conceived of as a perception of being important to communities, other individuals and to the self [61]. It has been linked to numerous aspects of mental health and wellbeing [61–67]. Equally, perceptions of not mattering, sometimes termed ‘anti-mattering’, have been found to be associated with an array of mental health problems, such as rumination, self-criticism, depression, perfectionism and perceived stigmatisation [64, 68–72]. However, the role of mattering and ‘anti-mattering’ in relation to suicidal experiences has been explored only to a small extent and mostly in undergraduate or college students [62, 73]. Our findings highlight ways in which participants saw themselves ranging from someone who felt irrelevant and inconsequential to someone who did matter to other people and did have a role. A small number of individuals experienced this as a change in their self-identity. This resonates with, and extends the extant literature by illustrating these contrasting perceptions, and the movement between them, which is clearly important for the development of therapeutic interventions. These findings also add to a growing and important research endeavour which is to better understand how people with severe mental health problems develop and sustain resilience to suicidal thoughts, plans, and acts [37, 38, 40–43, 45, 74, 75]. Perhaps unsurprisingly, substantial associations have been reported between mattering and resilience [61, 63, 76]. Focusing scientific and clinical innovations on understanding mattering and ‘anti-mattering’ in relation to suicidal experiences appears exceptionally beneficial.

An important idea across the interviews was feeling acknowledged and valued which contrasted with situations in which participants felt that they were not valued and were thought of as a burden. These impressions were formed from communications with friends, family, other people with mental health problems, and mental health professionals, for example, care co-ordinators and therapists. Not only were feelings of not being of value and being a burden linked to suicidal experiences, but they were also linked to beliefs about being undeserving of help for mental health problems. Indeed, some participants were concerned that if they did voice their psychotic and/or suicidal experiences, then that could be considered onerous, leading other people to seek social distance from them. This feeds into the importance of examining mattering in relation to suicidality but also melds with a large and expanding literature based on Thomas Joiner’s Inter-personal Psychological Theory (IPT) of Suicide [18, 20, 77]. Recent work has linked two strands of the IPT, that is, feeling a burden and ‘thwarted belongingness’, to perceptions of not mattering [78]. Our findings develop this further by showing how individuals can change these perceptions and also how mental health professionals can offer explicit clarity, reassurance and encouragement to clients about discussing psychosis and/or suicidality [79, 80]. In addition, this has implications for examining and countering attitudes which can appear stigmatising. Participants in the current study relayed experiences of feeling stigmatised from a diverse range of interactions, including those with family, friends, and mental health professionals. There is robust evidence documenting the negative effects of stigma on people with suicidal and psychotic experiences [81–84]. Furthermore, stigma can permeate clinical practice at a systems level, making it very difficult for individuals to feel able to talk openly about their suicidal thoughts and feelings without fear of being forced into compulsory detention which is clearly an issue for numerous stakeholders, including those involved with developing mental health policy [85].

A notable aspect of the findings was that dealing with the daily intensity of psychotic experiences, feeling suicidal, and for the majority of participants having additional debilitating mental health problems, meant that disconnection from other people felt, at times, desirable. This was despite some individuals recognising that not making contact with others is considered detrimental to mental health [25, 86, 87]. In some circumstances, enacting disconnection felt necessary for participants and something for which they had agency. In contrast, disconnection seemed particularly challenging when participants did not have agency, and felt abandoned or rejected. This resonates with qualitative work exploring psychotherapists’ perceptions of suicidal clients in which being able to form genuine connections with clients scarred by toxic relationships was central [80]. The sheer effort involved with getting through a day for someone with severe mental health problems, particularly psychosis together with suicidality, has been documented in the context of resilience [1]. The impact of anxiety on disconnectedness for such individuals has been only marginally investigated but is an area deserving of research attention because anxiety problems in people with psychosis are often overlooked but can be highly debilitating in their own right and/or because they interact with psychotic experiences [84, 88–90]. Although the effect of being bereaved and of friendships breaking up could be devastating, the pressure of feeling forced to have social contact could be equally as difficult which is an important point to consider in therapeutic settings. From a clinical perspective, it is important to be aware that suicidal experiences can escalate when close personal relationships change or are lost. Relatedly, for some participants, the experience of therapy coming to an end could seem abrupt. Even though therapy endings are usually carefully worked towards, especially with clients who have severe mental health problems [91], the current findings serve to underscore the overwhelming impact that therapy ending can have on individuals.

Central to the findings was the importance of talking about mental health problems, including suicidal thoughts and acts, even when that prospect was frightening. However, this ratification was not without ambivalence. One of the main reasons for which talking about suicide was a concern was because it might evoke ruminative processes, immerse individuals in traumatic past experiences, and subsequently amplify suicidality. This point has also been voiced in the suicide literature including by ethics committees [92]. A growing body of suicide research has adopted ways of monitoring the effect on participants of working on suicide-related research projects. In general, there have been more positive than negative participatory experiences [93–98] in both the short and longer term [99]. Furthermore, in previous studies participants expected that working with researchers on suicide could be somewhat upsetting [99]. This expectation was also articulated by participants in the current study which is reassuring.

A clear inhibitory factor in talking about mental health problems was that participants sometimes felt judged, not understood and/or misunderstood, and that they were given unhelpful advice in an overbearing manner, rather than being genuinely listened to. These experiences applied not only to talking with friends and family but also with mental health professionals. The ramifications of clients perceiving that they have not been listened to has been documented and voiced from different perspectives, including those of the Expert-By-Experience [100]. Indeed, friends and family members of people with psychosis and suicidal experiences also report a desire to be recognised and listened to by professionals, reflecting their wish to work collaboratively with health services in supporting their loved ones, especially in crisis situations (Marshall et al., 2022). The participants interviewed in the current study emphasised the importance of creating a safe space where the boundaries of confidentiality were clear from the outset.

A perception that talking would make no difference to the state of mind of someone who is feeling intensely suicidal was conveyed in the current study. A similar finding arose from work which used vignettes to explore thoughts and feelings about a protagonist who went from having suicidal thoughts to actively planning their suicide [45]. When the protagonist was at a stage of actively planning suicide, participants indicated that talking with those close to them would be ineffective but that the input of mental health professionals might be helpful. The same sentiments have been expressed in the current study. This represents an important educational and therapeutic target of relevance to both clients and mental health professionals which is that it is always important to communicate about suicidal thoughts and plans no matter how ‘advanced’ they may seem.

### Limitations and strengths

Three key limitations of this study warrant discussion. The first is that the topic guide explored mechanisms and pathways to suicidal experiences which involved interrelationships with friends, significant others, and relatives indirectly, rather than directly. That is, initially in interviews participants were encouraged to share the different ways that they had become suicidal in an open non-directive manner. It was anticipated that an indirect approach to exploring relationships with significant others would be less abrasive and more effective than direct questioning. Furthermore, the interviews did probe views about suicide-focussed therapy, meaning that perspectives about relationships with mental health professionals were invited.

Second, participants largely identified as having a White, Caucasian ethnicity. A considerable literature implicates stronger social support networks in minority populations [101–103]. In addition, Black and Asian Ethnic minorities can face substantial difficulties in getting help with their mental health difficulties including psychosis and harm to self [104, 105]. Thus, it is plausible that people from ethnic minority groups may have different perceptions of the role of interpersonal relationships compared to White individuals, and in particular different views and experiences of interactions with mental health professionals and talking therapies.

Third, although participants were recruited because they had recent suicidal and psychotic experiences, none were in an acute crisis, and all were under the care of services. Thus, the effects of intoxication from substances, thought disorder, agitation, and lack of treatment or monitoring were not explored.

Three main strengths should also be highlighted. The first strength is researcher triangulation which was used as a method to maximise the trustworthiness of the findings [47]. The current study benefitted from the perspectives of experts in suicide and severe mental health problems (PG) and qualitative research methods (SP) together with the wider research team comprising EBEs, psychiatrists, clinical psychologists, and nurses who contributed to regular discussion of evolving codes and themes, and analytical interpretations.

The second strength was that participants were selected to maximise richness and diversity of perspectives. Participants had all experienced non-affective psychosis and the final sample included a range of suicidal thought severity and lifetime suicide attempts. The participants, therefore, all had a high level of information power [106] yielding a data corpus that contained abundant, relevant and varied accounts of the interrelationships between severe mental health problems, suicidal experiences and interpersonal relationships.

The third strength was that all participants had a care-coordinator and approximately half of the participants had received a suicide-focussed talking therapy [13]. This meant that participants could be invited to relay their perceptions of talking with a mental health professional and/or their therapist about their experiences of relationships, suicide and psychosis.

### Clinical implications

There are three key implications for clinical practice. The first is to learn how to nurture in each client a sense that they do matter, and that they are valued, not only at a personal level but also more broadly to communities and within society. Relatedly, in clinical practice, a mental health service culture that focuses on risk assessment, rather than the management and acceptance of risk, sometimes makes clients feel that their problems are not acknowledged nor addressed with empathy, which in turn can make them disinclined, or even afraid, to communicate their experiences. This means that clear and explicit boundaries must be not only be communicated sensitively by practitioners, but a shared understanding endorsed by both practitioners and clients, especially with respect to confidentiality in a context of exploring suicidal thoughts and experiences, is also necessary. Second, involving peers with lived experience of suicidality and psychosis in both support initiatives and therapy delivery might counter perceptions of unwanted advice giving and overbearing attitudes whilst fostering genuine understanding and bolstering support networks. Clearly, excellent and acceptable support frameworks for peer workers need to be in place in addition to training and effective integration in multidisciplinary mental health teams with a focus on suicidal experiences. Third, it could be beneficial to actively and collaboratively involve family and friends not only as central members of the mental health teams of clients living with psychosis and suicidality, but more directly in therapy work. One of the participants who we interviewed made this suggestion whilst also understanding the complexities that this can introduce.

## Conclusions

Participants portrayed ways in which psychosis, suicidality and interpersonal relationships were intertwined. Being involved in personal relationships with other people, including friends, relatives and significant others, had the effect of countering suicidal experiences but could also impede the ways that people lived with their mental health problems. This balance between hindering and helping extended to mental health professionals. It was notable that individuals portrayed a dynamic between feeling that their lives were of little consequence, to feeling acknowledged and valued. This is fundamentally important for two reasons. First, it illustrates ways in which people with psychosis can develop and maintain psychological resilience to mental health problems of which having suicidal experiences are frequent. Understanding resilience is an expanding area in suicide research [37, 38, 45]. Second, it provides a basis for developing effective suicide-focussed therapeutic approaches which build on the insights of people with lived experiences of suicidality and psychosis.

## Data Availability

The raw datasets generated and analysed during the current study are not publicly available as participants did not provide consent for this. De-identified data are available from the corresponding author upon reasonable request.
